# Olive Oil/Pluronic Oleogels for Skin Delivery of Quercetin: In Vitro Characterization and Ex Vivo Skin Permeability

**DOI:** 10.3390/polym13111808

**Published:** 2021-05-31

**Authors:** Mohammed Elmowafy, Arafa Musa, Taghreed S. Alnusaire, Khaled Shalaby, Maged M. A. Fouda, Ayman Salama, Mohammad M. Al-Sanea, Mohamed A. Abdelgawad, Mohammed Gamal, Shahinaze A. Fouad

**Affiliations:** 1 Department of Pharmaceutics, College of Pharmacy, Jouf University, Sakaka 72341, Saudi Arabia; khshalabi@ju.edu.sa; 2 Department of Pharmaceutics and Industrial Pharmacy, Faculty of Pharmacy (Boys), Al-Azhar University, Nasr City, Cairo 11765, Egypt; ayman1grawan@gmail.com; 3 Department of Pharmacognosy, College of Pharmacy, Jouf University, Sakaka 72341, Saudi Arabia; 4 Department of Pharmacognosy, Faculty of Pharmacy, Al-Azhar University, Cairo 11371, Egypt; 5 Biology Department, College of Science, Jouf University, Sakaka 72341, Saudi Arabia; tasalnosairi@ju.edu.sa (T.S.A.); mmfouda@ju.edu.sa (M.M.A.F.); 6 Department of Zoology, Faculty of Science, Al-Azhar University, Assiut 71511, Egypt; 7 Department of Pharmaceutics, Faculty of Pharmacy, University of Tabuk, Tabuk 47711, Saudi Arabia; 8 Department of Pharmaceutical Chemistry, College of Pharmacy, Jouf University, Sakaka 72341, Saudi Arabia; mmalsanea@ju.edu.sa (M.M.A.-S.); mohamedabdelwahab976@yahoo.com (M.A.A.); 9 Department of Pharmaceutical Organic Chemistry, Faculty of Pharmacy, Beni-Suef University, Beni-Suef 62514, Egypt; 10 Pharmaceutical Analytical Chemistry Department, Faculty of Pharmacy, Beni-Suef University, Beni-Suef 62514, Egypt; mgmohamed@ju.edu.sa; 11 Department of Pharmaceutics, Faculty of Pharmacy, Ahram Canadian University, Giza 14756, Egypt; shahinazeamry9@gmail.com

**Keywords:** oleogel, skin delivery, quercetin, olive oil

## Abstract

The main objective of this study was to prepare and characterize oleogel as potential carrier for quercetin skin delivery. The formulations were prepared by adding olive oil (5–30%) to Pluronic F127 hydrogel and were evaluated for particle size, zeta potential, viscosity in vitro quercetin release and stability, and were compared with that of Pluronic F127 hydrogel. The selected formulation was characterized for its interaction possibility, ex vivo skin permeation and skin histological changes and safety. The particle sizes ranged from 345.3 ± 5.3 nm to 401.5 ± 2.8 nm, and possessed negative charges. The viscosities of the formulations were found in the range of 6367–4823 cps with inverse proportionality to olive oil percentage while the higher percentages showed higher quercetin release. Percentages of 25% and 30% olive oil showed instability pattern under the conditions of accelerated stability studies. Differential scanning calorimetry verified the existence of quercetin in micellar aggregation and the network in the case of hydrogel and oleogel respectively. Ex vivo skin permeation showed an improved skin permeation of quercetin when 20% olive oil containing oleogel was used. Skin histology after 10 days of application showed stratum corneum disruption and good safety profile. Based on these findings, the proposed oleogel containing 20% olive oil denotes a potential carrier for topical delivery of quercetin.

## 1. Introduction

The skin is the largest organ of human body and acts as biological barrier which protects the body any external threatening hazards. The external hazardous stimuli may be in the form of ultraviolet and visible radiation, microbial invaders, ionizing radiation and oxidants (reactive oxygen species; ROS) [1,2]. However, self-defending action specially against ROS was reported by the antioxidant effect of endogenous superoxide dismutase, metallothionein and melanin [3]. Nevertheless, if the oxidative stress of ROS overweighs skin self-protective effect, skin harms may take place such as lipid peroxidation, DNA breakage, tumor promotion [4] and, hence, skin aging. Therefore, assistance of the skin self-protective systems by exogenous antioxidants could preclude ROS-induced cuteneous damage. Polyphenolic compounds, including flavonoids, are well known as powerful antioxidants and are generally used for topical use as a protectants against natural skin damage (as photo-ageing), skin cancer prevention and skin cosmetic-care [5,6,7].

Flavonoids, a class of phenolic compounds, are considered powerful antioxidants. Their action depends upon several mechanisms including chain-breaking inhibition of the peroxidation process, scavenging free radicals and iron chelating effect leading to antilipoperoxidative action [8]. However, their actions against lipid peroxidation of the biomembranes rely on their molecular structure and capability to interact and infiltrate the lipid bilayer [9]. Among those, quercetin (QT; 3,3’,4’,5,7-pentahydroxyflavone) is considered as one of the most potent natural antioxidants [10]. It also has anti-inflammatory, anticarcinogenic, antibacterial and antiviral effects [11]. However, despite the documented QT beneficial therapeutic actions, its limited aqueous solubility (~2 µg/mL) hinders its skin absorption and effectiveness [12]. Several approaches have been implemented seeking for enhancing QT skin permeability, including combined microneedles and lipid microparticles [13], sodium hyaluronate-chitosan multilayered liposome [14], lipid-based nanosystems [15] and essential oil-based microemulsions [16].

Oleogels (organogels) are semi-solid systems prepared by the encapsulation of organic liquids, such as vegetable oils, in a three-dimensional polymer network [17]. They possess unique characteristics such as stability which is attributed to physical interactions such as hydrogen bonding, Van der Waals forces or chemical interactions as covalent bonds [18], biocompatibility, non-irritating nature and easiness of fabrication. Therefore, thanks to their core nature, oleogels represent a potential system possessing outstanding characteristics for skin delivery. They have been mostly formulated and studied for the cutaneous administration of active pharmaceutical ingredients (APIs) such as sumatriptan [19], melatonin [20], silymarin [21] and mefenamic acid [22].

Therefore, it seems quite justified to develop oleogels from Pluronic F127 and different concentrations of olive oil, one of the natural oils that enhance skin permeation [23], to be used as a platform for skin delivery of QT at concentrations of 5% to 30% (*w*/*w*) in relation to olive oil. Formulations were characterized for particle size, zeta potential, viscosity in vitro QT release and stability, and compared with these findings of Pluronic F127 hydrogel. The findings were utilized to select the most appropriate composition in the development of cutaneous QT delivery.

## 2. Materials and Methods

### 2.1. Materials

Quercetin (QT) and pluronic F127 were purchased from Sigma Aldrich (India). Olive oil (extra virgin) was purchased from Loba Chemie (India). All other chemicals were in analytical grades.

### 2.2. Preparation of QT-Loaded Pluronic F127/Olive Oil Oleogels

All the batches were fabricated in two steps. Firstly, the aqueous phase was prepared dispersing *Pluronic* *F127* (slowly to avoid clumping of the particles; 20% *w*/*v*) in phosphate buffer under magnetic stirring. After obtaining a homogeneous hydrogel the solution was kept at 4 C overnight to obtain transparent hydrogel. Secondly, QT was solubilized in olive oil under magnetic stirring. QT oily dispersion (in different ratios) was added into aqueous solution (in 1:4 ratio respectively) with aid of homogenization (yellow Line, GmbH, Germany) at 5000 rpm for 10 min. The compositions of the batches are defined in Table 1 with 0.2% of QT in the final preparation.

### 2.3. Particle Size and Zeta Potential

The prepared QT-loaded pluronic F127–olive oil oleogels were diluted 200 times with double distilled water, vortexed (Dragonlab cortex, Beijing, China) and kept in bath sonicator (specification) for 5 min. The dispersions were analyzed for particle size, polydispersity index (PDI) and surface using Zetasizer analyzer (Malvern Zetasizer Nano ZS90, Worcestershire, UK) at room temperature. All measurements were performed in triplicate.

### 2.4. QT Content

An accurately weighed 0.5 g of each batch was dissolved in 100 mL of absolute ethyl alcohol, sonicated and filtered to get rid of the residues. Drug free formulation was used as zero drug content formulation and subjected to the same procedure. QT level was measured by UV-visible spectrophotometer (Genesys 10S UV-VIS, Thermo Scientific, Shanghai, China) at 372 nm [24].

### 2.5. Organoleptic Characteristics

The prepared formulations were investigated for color, odor, softness, appearance and ease of application [25].

### 2.6. pH Determination

The prepared formulations were investigated for pH using a pH-meter (Hanna Instruments, Woonsocket, RI, USA) by direct dipping of the electrode into the formulation. All the measurements were made in triplicate.

### 2.7. Viscosity Measurement

The prepared formulations were investigated for viscosity by Brookfield rheometer (DV3T Rheometer, Middleboro, MA, USA) using spindle no. 7 with a speed of 20 rpm at room temperature. All the measurements were made in triplicate.

### 2.8. In Vitro QT Release

The in vitro QT release from different batches was performed using dialysis bag method. Accurately weighed 100 mg of oleogels was put inside cellulose acetate dialysis bags (M. wt. cut off: 12,000–14,000 Da, Livingstone, Australia). The bags were dipped into 200 mL of receptor solution consisting of PBS (pH 7.4) and absolute ethyl alcohol (70:30, *v*/*v* respectively) to increase the solubility of QT and maintain the sink conditions. The receptor solution was kept stirred at 50 rpm and temperature was maintained at 37 °C. At predetermined time intervals (30, 60, 120, 240, 360, 480, 600, 720 and 1440 min), 1 mL was withdrawn from the receptor solution to determine QT concentration spectrophotometerically with replenishment of receptor solution with the same volume of fresh media. All samples were measured in triplicate.

### 2.9. Accelerated Stability Test

Accelerated stability studies were performed using freeze–thaw cycles. Briefly, the prepared formulations were incubated at 70 °C for 15 min then transferred to −20 °C for 15 min. This cycle was done for five times. Afterwards, the batches were checked for phase separation or any other instability features.

### 2.10. Differential Scanning Calorimetry (DSC)

The thermal analyses of QT, Pluronic F127, F1 and selected formulation (F4) were conducted by a differential scanning calorimeter (DSC3, Mettler Toledo, Greifensee, Switzerland). Six milligrams of each sample were separately placed in an aluminum pan. Heating scan rate was adjusted 20 °C/min and temperature was recorded between 25 °C and 350 °C. Data were assessed with STAR^e^ 15.00 software.

### 2.11. Fourier Transform-Infrared Spectroscopy

Fourier transform infrared spectroscopy (ATR-FTIR; Thermo Scientific) was used to examine system compatibility based on wavelength function group. QT, Pluronic F127, olive oil, F1 and F4 were analyzed between 4400 and 400 cm^−1^ in transmission mode.

### 2.12. Ex Vivo Skin Permeation

The animals used during the experiments were obtained from the Animal House Colony of College of Pharmacy, Jouf University. The study was performed according to ethical procedures and policies of Jouf University (Approval code; LCBE6/5/42), which verified the laboratory animal care obeyed the guidelines of the Declaration of Helsinki and the Guiding Principles for the Care and Use of Laboratory Animals. The procedures also followed the National Institutes of Health Guide for the Care and Use of Laboratory Animals (NIH Publication No. 8023, revised 1978).

The skin permeation of QT was studied using Franz diffusion cells (Logan DHC-6T Dry Heat Transdermal System) equipped with temperature control, autosampler, and reservoir replacement automatic system. The temperature was maintained at 37 ± 1 °C. After sacrificing the animals, the hairs on the dorsal side of animals were removed. The skin was harvested, freed of adhering fat layers and mounted on Franz diffusion cells. The receptor compartment was filled with twelve milliliters of a (PBS)/absolute ethyl alcohol (pH = 7.4, 70:30) with 0.11% (*w*/*v*) formaldehyde as a preservative [26]. Freshly excised full thickness rat skin was cut into pieces with dimensions relevant to cell diffusion area (1.13 cm^2^) at which stratum corneum (SC) faced the formulation. Ten mg of various formulations were applied onto the skin in the donor compartment. At 30, 60, 120, 180, 240, 300 and 360 min time points, aliquots of 0.5 mL were withdrawn to be assayed spectrophotometerically. The same volume of fresh receptor medium was added to receptor compartment to maintain constant receptor volume.

### 2.13. Permeation Kinetics

After plotting of the mean cumulative QT permeated against time points, different kinetic models were applied to check the best fitting model depending on the correlation coefficient (*r*^2^). In that regard, zero-order model, first-order model, Higuchi’s model and Korsmeyer–Peppas (KP) model were applied.

### 2.14. Histological Changes and Safety

In this section we investigated the histological changes and safety of the formulations through 10 days of daily application (at days 3, 7 and 10). At predetermined days, the application areas were visually checked for any noticeable changes such as erythema. The average erythemal scores were noted (ranging from zero to four) based upon the degree of redness. Afterwards, the animals were sacrificed, areas of application were excised, fixed with 10% formalin solution and stained with H&E for the histopathological investigation.

### 2.15. Statistical Analysis

In this study, the results are presented as means ± standard deviations (SD). The statistical analysis was performed using one-way ANOVA and means were compared using Tukey’s multiple comparison testing with GraphPad Prism v.5. Software. *p* < 0.05 was considered a significant difference.

## 3. Results and Discussion

### 3.1. Preparation and Formulation of QT Loaded Olive Oil/Pluronic Oleogels

Among the various drug delivery systems, oleogels were selected as promising system to deliver QT to the skin. Oleogels are potential candidates for dermocosmetics and personal care products [27,28,29]. Table 1 shows the constitutions of seven different QT loaded hydrogel and oleogels formulated for the current study, which were designed on the basis of olive oil percentage (5–30%) aiming to study the effect of the these percentages on different physicochemical characteristics and skin permeability.

### 3.2. Particle Size and Zeta Potential

The particle size and zeta potential are key elements for estimation of the stability of the colloidal delivery system during storage [30]. Additionally, both parameters also were reported to affect cutaneous drug delivery across skin barrier [31,32]. Table 1 shows the particle size and zeta potential of all investigated formulations. It was clear that the particle size they in nanosized range (in range from 345.3 ± 5.3 nm to 401.5 ± 2.8 nm) while the zeta potential was negative and in range from −17.2 ± 3.1 mV to −15.1 ± 2.3 mV. Olive oil addition (by any percentage) did not significantly (*p* > 0.05) influence both parameters.

### 3.3. QT Content and Organoleptic Characteristics

The drug content of the prepared oleogels was found to be high and uniform in range from 95.1 ± 4.6% to 97.6 ± 8.5, as shown in Table 1. It is clear that addition of olive oil at any level did not significantly (*p* < 0.05) influence the drug content. This behavior was attributed to the fact that Pluronic formed homogenous distribution of QT throughout the matrix and could incorporate most of the amount of QT added [30]. On the other hand, olive oil/Pluronic could not be confirmed at this step and would be checked by further experiments. Changes in organoleptic properties based on the concentration of olive oil are depicted in Table 2. Under the experimental conditions, all the prepared formulations were grittiness free with a smooth feel and did not show phase separation of exudates. All formulations were non-greasy except F6 (30% olive oil content) which showed less greasiness. The consistencies of the formulations were decreased with the increase in olive oil concentration. The color of olive oil free formulation (F1) was brownish while olive oil-containing formulations were light yellow.

### 3.4. pH and Viscosity Measurements

The pH values of the Pluronic F127 hydrogel and oleogels were slightly acidic and found to be in the range of 6.6 ± 0.8 and 5.8 ± 0.6 (Table 2). It is clear that the value of pH decreased with the increase in olive oil concentration. Acidity rise may be ascribed to the content of fatty acids. It was reported that olive oil contains a high percentage of monounsaturated fatty acids (MUFA) which are accounting about 85% of its composition. The main fatty acids are oleic acid (C18:1), which might range between 70% and 85%, and other fatty acids such as linoleic or palmitoleic acid [33]. Generally, this pH range is safe and expected to induce no dermal irritation reactions [34]. Viscosity profiles (Table 2) indicated that the viscosity of the hydrogel was higher than oleogels and higher concentration of olive oil produced lower viscosity. The viscosities of the hydrogel and oleogels were found in the range of 6367–4823 cps. In hydrogel, adding Pluronic F127 in critical micelle concentration into water led to formation of micelles [35] and the viscosity raised. It was reported that aqueous solution of Pluronic F127 at 20–40% concentration resulted in a face-centered cubic structure of micelle [36] and hence high viscosity [37]. As adding olive oil to Pluronic decreased the percentage of solids in relation to liquid, it resulted in consistency lowering [19]. Additionally, increasing the percentage of olive oil from 5% to 30% could decrease the water percentage in the formulation resulting in disturbance in the entanglement of tubular micelles. This result is in good accordance with the previous study [25].

### 3.5. In Vitro Release Studies

**Figure 1:**
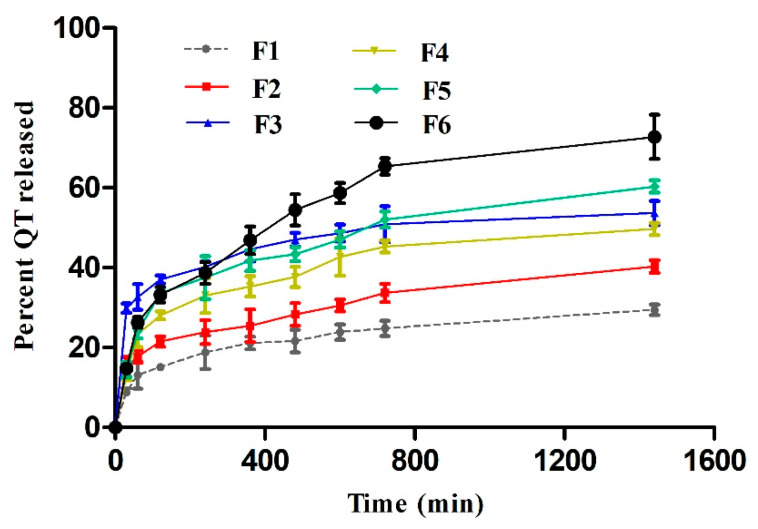
In vitro release profiles of QT from different formulations (mean values ± SD, n = 3).

In vitro QT release from different formulations was assessed over a period of 24 h. As depicted in Figure 1, the cumulative percentages of QT released were plotted against predetermined time intervals. The results showed that the highest percentage of QT release (72.7%) after 24 h was observed from F6; the formula which contained the highest percentage of olive oil while the lowest release percentage (29.4%) was observed in olive oil free Pluronic F127 hydrogel. By another way, the release of QT was olive oil concentration dependent; the higher the olive oil concentration, the higher QT release percentage. It is suggested that the viscosity played a crucial in controlling drug release [19]. As mentioned above, olive oil decreased the water percentage in the formulation resulting in disturbance in the entanglement of tubular micelles then lowering the formulation viscosity. On the other hand, 20% Pluronic F127 is a thermogelling system and can form gels above its critical micelle concentration (CMC). In addition, keeping Pluronic F127 solution for 24 h at 37 °C triggered gelation and viscosity elevation owing to dehydration of polypropylene oxide block in the polymer micelles’ extra aggregation [38]. This pattern encouraged us to measure the viscosity after performing the release experiment. As expected, F1 hydrogel viscosity was significantly (*p* < 0.05) increased to 8564 ± 69 cp while the other formulations were not significantly changed. This in turn suggested the micellisation disturbance in the presence of olive oil, which needed further assessment. Anyhow, shifting into higher viscosities extended the release of the active drug from the gelling matrix.

### 3.6. Accelerated Stability Test

Accelerated stability testing of the prepared formulations was performed by freeze–thaw cycles to expect the long-term stability of the prepared oleogels. The formulations were checked for any signs of instability during the five cycles. Two oleogels (F5 and F6) were unable to preserve their structural integrity and phase separation was observed at the end of the period while the other formulations maintained the stability features. F5 and F6 were of 25% and 30% olive oil contents, respectively. Although it has been reported that the gelation of the oil helps prevent the migration of oils in the pharmaceutical products and thus improves the shelf-life stability of the products [39], higher concentrations of olive oil provoked product instability. Based on the results described above, F4 was selected for the subsequent studies as it contained the highest percentage of olive oil with good release and stability behavior.

### 3.7. Thermal Analysis

**Figure 2:**
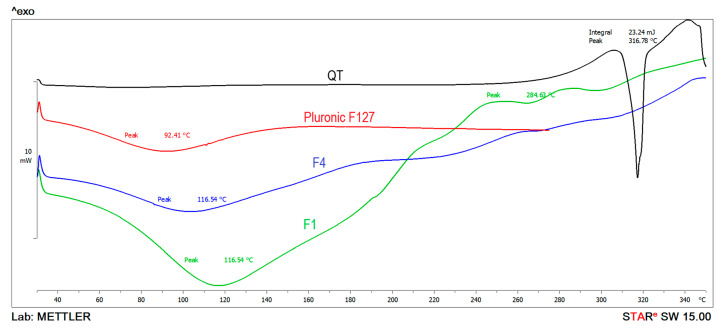
DSC thermograms of of QT, Pluronic F127, F4 and F1.

The thermal behavior of pure QT, Pluronic F127, F1 and F4 was appraised by evaluating DSC thermograms. However, olive oil (liquid state) could not be registered using the defined temperatures and analytical circumstances [40]. Figure 2 shows the thermograms of samples under investigation. Pure QT showed main sharp endothermic at 316 °C (Figure 2) confirming its crystallinity. Pluronic F127 showed the peak at 92 °C. For F1 and F4, broad peaks at 116 °C were observed (peak onset at 81.7 °C) which might be due to overlapped typical Pluronic F127 peak with another peak representing the loss of moisture from hydrogel and oleogel. Furthermore, the intensity of the peak in the case of F1 was more pronounced in F1 than that in F4, which was suggested to be due to higher water content in hydrogel (F1) when compared to oleogel (F4) [41]. Interestingly, Hydrogel (F1) showed faint endothermic peak at 284 °C while oleogel (F4) theromgram was deprived from such peak. This behavior of F1 was suggested to be due to breakdown of the reverse micellar structures [42]. This could partly explain the micellisation interference effect of olive oil and consequent lower consistency of oleogels when compared to hydrogel. On the other hand, disappearance of QT main peak could be due to incorporation of micelles in the case of F1 and presence of amorphous oil soluble form in the case of F4.

### 3.8. FT-IR

**Figure 3:**
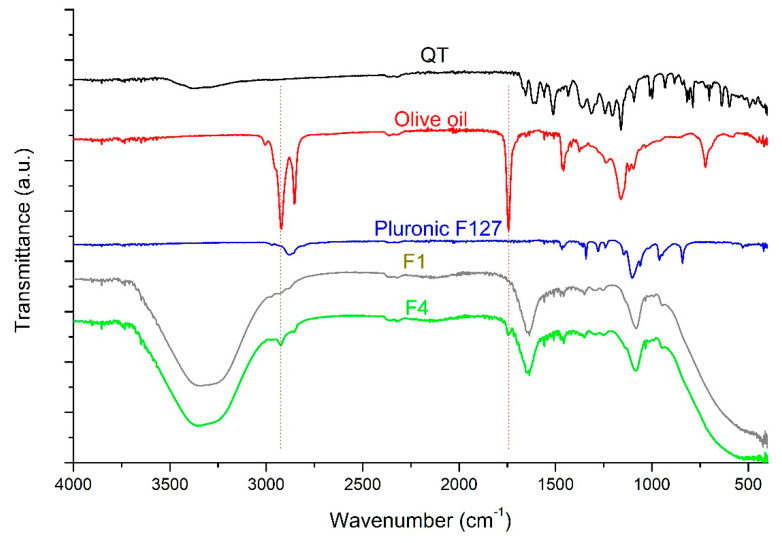
FT-IR spectra of QT, olive oil, Pluronic F127, F1 and F4.

An interaction between the components was studied using FT-IR spectroscopy in transmittance mode. Figure 3 represents fingerprint region spectra of QT, olive oil, Pluronic F127, F1 and F4. The FT-IR spectrum of raw QT displayed main peaks at 3379 cm^−1^ (revealing free hydroxyl bond vibration), 1654 cm^−1^ (revealing band stretching vibration of carbonyl group), 1618 cm^−1^ (revealing C–C stretching vibration of phenyl ring), 1512 cm^−1^ (revealing aromatic group), at 1315 cm^−1^, 1160 cm^−1^ (revealing C–O–C vibration), and at 996 cm^−1^ (revealing aromatic C–H group) [43]. Olive oil spectrum showed strong band absorption peaks at 2920 cm^−1^ and 2854 cm^−1^ (revealing C–H stretching vibration of methylene and methyl groups respectively), 1741 cm^−1^ (revealing carbonyl double bond stretching vibration) [44]. Pluronic F127 spectrum exhibited at 2824 cm^−1^ (revealing C-H stretching), 1281 cm^−1^ and 1239 cm^−1^ (revealing the stretching of C–O–C bonds) and 1105 cm^−1^, (revealing C-O stretching) [45]. In the spectra of F1 and F4, they presented similarly except for the appearance of new peaks in F4 spectrum at 2920 cm^−1^ and 1753 cm^−1^ correspond to the typical stretching vibrations of olive oil (dashed lines). On the other hand, most of the QT characteristic peaks disappeared in both F1 and F4 with slight shifting in few absorption peaks. This might be attributed to the alteration in the chemical environment around the components of the oleogels. Additionally, broader peaks (when compared to raw QT) at 3332 cm^−1^ and 3348 cm^−1^ were observed in spectra of F1 and F4, respectively, which supposed the occurrence of intramolecular/intermolecular hydrogen bonding among the oleogel constituents [46]. It was also observed that no additional peaks in F1 and F4 spectra which might be ascribed to lower concentration of QT when compared to oleogel forming matrix and hence QT peaks were lessened owing to the strong peaks of the other constituents [41].

### 3.9. Ex Vivo Skin Permeation

**Figure 4:**
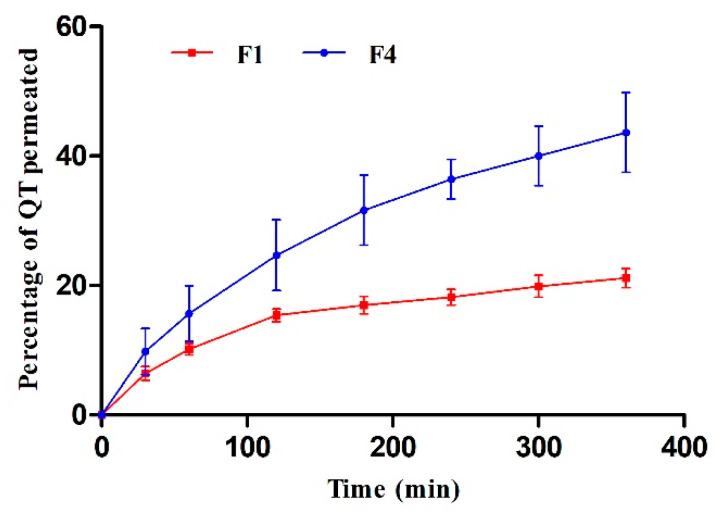
Representative graphs of the cumulative amounts of QT permeated through full thickness skin samples versus time for F1 and F4 (mean values ± SD, n = 3).

**Figure 5:**
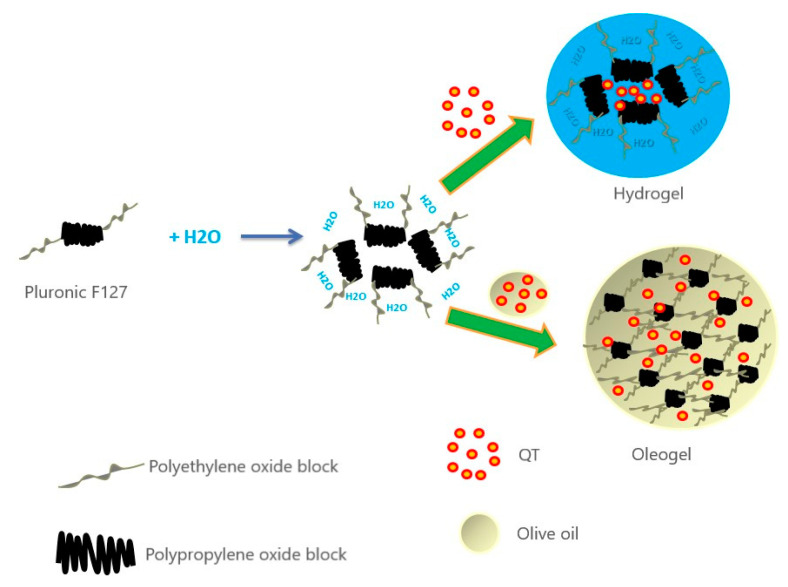
Schematic representation of the mechanism of formation of pluronic olive oil oleogels.

In vitro permeation profiles of F1 and F4 were achieved using Franz diffusion cells to appraise the influence of both formulations on the diffusion of QT through the full-thickness skin under a non-occlusive application. Figure 4 shows the percentage of the amount of QT that permeated through the skin versus predetermined time points. The cumulative percentages of QT which permeated through the skin into receptor compartment from F1 and F4 after 6 h were 21.2 ± 1.4% and 43.6 ± 6.2%, respectively. It is obvious that QT permeation from F4 was about two times higher than F1. Formulation/skin interaction was particularly related to its structure and components. F1 (hydrogel) is structurally aggregative micelles encapsulating the hydrophobic drug in the hydrophobic block core of Pluronic F127 (polypropylene oxide) [47] while F4 (oleogel) is self-sustained thermo-reversible network (represented by Pluronic F127) which immobilizes an organic compound (represented by olive oil) [48] (Figure 5). In the case of F1, QT escaping was confined by aqueous external surroundings and high consistency, while escaping from F4 was higher due to easier release and low consistency of the matrix when compared to F1. Regarding the components, Pluronic F127 is surface active agent and can interact with the skin, causing disturbance in the lipid barrier in the horny layer and increase skin permeability. Adding olive oil (oleic acid rich oil) to Pluronic F127 could trigger the skin permeability through disrupting the cellular arrangement of the stratum corneum irreversibly and thereby allowed better penetration through the different skin layers [49]. Additionally, the miscibility/mixing of olive oil with stratum corneum lipids could also promote skin penetration. Furthermore, the packing pattern of fatty acids altered the fluidity of skin lipid structure and assisted QT skin absorption [50].

Table 3 shows the kinetic parameters obtained by fitting the release pattern to different models. From the results we can say that the model that F1 and F4 followed the Korsmeyer–Peppas model as they displayed higher r^2^ values of 0.986 and 0.996 respectively, which are close to 1.0. F1 showed a value of n = 0.413 (less than 0.5), indicating that release kinetics of QT from the hydrogel containing the highest amount of particles was controlled by Fickian diffusion. On the other hand, F4 showed a value of n = 0.562 (intermediate value between 0.50 and 1.00) indicating an anomalous behavior (non-Fickian kinetics corresponding to the phenomena of diffusion and relaxation of the polymer chains) [51].

### 3.10. Histopathology

**Figure 6:**
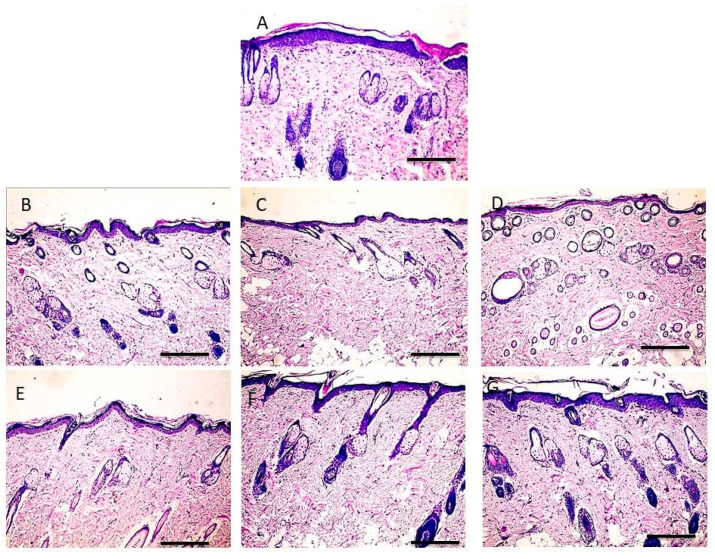
Microscopic pictures of H&E-stained skin sections from control rats (**A**) and rats exposed to F1 for 3 days (**B**), 7 days (**C**) and 10 days (**D**) and F4 for 3 days (**E**), 7 days (**F**) and 10 days (**G**) showing no structural damage in dermal elements (magnification 100×, bar 100).

Skin histopathology was implemented to study the effects of F1 and F4 on the structure of rat skin. Figure 6A shows the histopathology of control untreated skin which exhibited a clear delineation between the epidermis and dermis, with a compact stratum corneum and well-arranged corneocytes. However, application of F1 (Figure 6B–D) and F4 (Figure 6E–G) for 10 consecutive days did not induce significant changes at the level of dermis. The difference was clearly induced at the level of epidermis. F4 showed more predominant disruption of the stratum corneum integrity and swelling when compared to F1. This increase in the thickness of the stratum corneum was attributed occlusive effect of olive oil which could increase skin hydration by precluding trans-epidermal water loss, leading to swelling effect [52]. This in turn could weaken the core barrier of the stratum corneum and improve the skin delivery, which was ascertained by ex vivo skin permeation studies.

## 4. Conclusions

In the present work, oleogels containing different amounts of olive oil were formulated and characterized. The prepared formulation possessed particle size in nano-range and negative surface charges. The viscosities of the formulations were found in an inverse proportionality to olive oil percentage while the higher percentages showed higher quercetin release. Percentages of 25% and 30% olive oil showed instability pattern under the conditions of accelerated stability studies. DSC results indicated that QT was embedded into in micellar aggregation and the polymeric network in the case of hydrogel and oleogel respectively. Oleogel containing 20% olive oil showed an improved skin permeation presenting more than 2-fold when compared to hydrogel. It also showed good safety profile on the skin without showing significant dermatotoxicity. Overall results suggest that 20% olive oil in 20% Pluronic F127 oleogel is a good candidate for improving topical QT skin delivery. The potential of the fabricated system could be further investigated by carrying out clinical trials and scale-up studies.

## Figures and Tables

**Table 1:** Compositions, mean particle size, zeta potential and drug content % (means ± SD, n = 3) of different formulations.

Code	Olive Oil %	Pluronic F127 %	Particle Size (nm)	Zeta Potential (mV)	Drug Content %
F1	-	20	345.3 ± 5.3	−15.1 ± 2.3	96.5 ± 2.8
F2	5	20	375.9 ± 5.7	−19.7 ± 3.7	95.1 ± 4.6
F3	10	20	389.4 ± 3.8	−19.2 ± 2.5	95.7 ± 7.3
F4	15	20	372.8 ± 7.9	−16.1 ± 3.3	96.0 ± 9.2
F5	20	20	369.8 ± 8.5	−17.3 ± 6.1	97.1 ± 2.8
F6	25	20	401.5 ± 2.8	−15.5 ± 1.2	96.1 ± 4.9
F7	30	20	392.8 ± 9.7	−17.2 ± 3.1	97.6 ± 8.5

**Table 2:** Organoleptic characteristics, pH and viscosity (means ± SD, n = 3) of different formulations.

Code	Organoleptic Characteristics					pH	Viscosity (cp)
	Phase Separation	Greasiness	Grittiness	Consistency	Exudate		
F1	None	None	None	Viscous, spreadable gel	Nil	6.6 ± 0.8	6367 ± 28
F2	None	None	None	Viscous, spreadable gel	Nil	6.5 ± 0.2	6241 ± 34
F3	None	None	None	Less viscous, spreadable gel	Nil	6.3 ± 0.7	5836 ± 27
F4	None	None	None	Less viscous, spreadable gel	Nil	6.2 ± 0.4	5633 ± 49
F5	None	None	None	Thin, fluid	Nil	6.0 ± 0.4	5289 ± 23
F6	None	None	None	Thin, fluid	Nil	6.0 ± 1.1	5003 ± 36
F7	None	Less observable	None	More thin and fluid	Nil	5.8 ± 0.6	4823 ± 29

**Table 3:** Kinetic parameters for the ex vivo skin permeation of QT from formulations F1 and F4.

Code	Zero Order Kinetics		First Order Kinetics		Higuchi Model		KP Model	
	r^2^	K_0_	r^2^	K_1_	r^2^	K_H_	r^2^	n
F1	0.630	0.07	0.697	0.001	0.973	1.19	0.986	0.413
F2	0.855	0.14	0.934	0.002	0.992	2.29	0.996	0.562

## Data Availability

Not applicable.
